# Uptake characteristics of MRI and CT contrast agents in 3D printing materials for vascular phantoms

**DOI:** 10.1186/s41205-025-00309-3

**Published:** 2025-11-28

**Authors:** Annika C. Dell, Celina Wist, Hanna Grasshoff, Malte M. Sieren, Maria-Josephina Buhné, Constantin Schareck, Patricia Ulloa, Thorsten M. Buzug, Roman Kloeckner, Jörg Barkhausen, Alex Frydrychowicz, Thomas Friedrich, Franz Wegner

**Affiliations:** 1 Fraunhofer IMTE, Fraunhofer Research Institution for Individualized and Cell-Based Medical Engineering, Lübeck, Germany; 2 Clinic of Rheumatology and Clinical Immunology, University Hospital Schleswig-Holstein, Lübeck, Germany; 3 Institute of Radiology and Nuclear Medicine, University Hospital Schleswig-Holstein, Lübeck, Germany; 4 Institute of Interventional Radiology, University Hospital Schleswig-Holstein, Lübeck, Germany; 5 Institute of Neuroradiology, University Hospital Schleswig-Holstein, Lübeck, Germany

**Keywords:** 3D printing, 3D printing materials, Compliant vascular models, Vasculature, Vascular model, MRI, CT, Contrast agent, Vascular phantom

## Abstract

**Background:**

To determine the reusability and robustness of three 3D-printing materials for anatomical vessels by evaluating their contrast agent uptake characteristics throughout different timeframes.

**Methods:**

The tested samples were 3D-printed cylindrical samples that have the same diameter and wall thickness as a healthy adult aorta. Three different materials of varying degrees of Shore hardness were used to print these samples on a Stratasys J850 Prime PolyJet 3D printer (VeroClear, Agilus, and an Agilus/VeroClear mixture). Each sample was filled with one of three contrast agent dilutions or a control solution. Samples remained filled with their respective solution for one week, one day, or one hour. Computed tomography (CT) and magnetic resonance (MR) images were taken of all 54 samples. The CT and MR images were evaluated to determine the diameters of the samples, as well as the radiodensity/signal intensity of the samples. An Intraclass Correlation Coefficient (ICC) was calculated to determine the degree of measurement variation between the investigators. Sample mass increase was determined by weighing samples before and after exposure to the solutions. A generalized linear mixed model (GLMM) was used to evaluate the contrast agent uptake behavior of the materials based on CT and MR imaging data.

**Results:**

A small relative mass increase in the 3D-printed materials was noted: VeroClear showed mass increases of between 1.1% and 2.5%, which is in line with the respective data sheet. Agilus showed mass increases of 2.9% to 4.4%. In CT images, very small, but statistically significant effects were detected for VeroClear in the measured diameters (*t* = −2.31, *p* = 0.02) and the signal intensity (*t* = 3.40, *p* < 0.001). All other tested material combinations revealed no significant effects in comparison to the reference sample.

**Conclusions:**

This study suggests that the tested materials using the Stratasys J850 3D printer can produce structures that do not absorb clinically detectable amounts of CT or MR imaging contrast agent solutions. Thus, the tested materials are suitable for the 3D printing of vascular phantoms that are filled with contrast agent solutions and can be reused in the time periods evaluated.

## Background

Cardiovascular diseases are the primary cause of death globally, giving rise to a need for realistic vascular models for endovascular device development and operator training [1]. Currently, different types of 3D-printed vascular models are used in clinical medicine and research [2–5], and their suitability for different clinical applications has been graded using guidelines [6, 7]. Specifically, compliant 3D-printed models are desirable, as they allow for both anthropomorphic geometry through segmentation of patient data, as well as realistic distensibility that emulates the flexible nature of vascular structures [8, 9]. Animal and cadaver models are often used for training or in vivo device trials; however, these options have ethical complications, high costs, limited availability, and do not always accurately represent the desired anatomy or pathology. 3D-printed benchtop anatomical models allow for reproducibility with negligible variability, longer shelf-lives, adaptation to a specific experiment, swift manufacturing, cost-effective solutions, and the circumvention of animal or cadaveric models [10–12].

Perfused vessel phantoms that use pumps in closed circulation systems to emulate blood flow within the vascular models are of interest, as these physiological pressurized conditions allow for increased realism for device testing and training applications [13]. Perfused vascular models can be used in combination with various imaging modalities, including X-ray, computed tomography (CT), and magnetic resonance (MR) for imaging experiments and simulation of endovascular procedures [14–16]. To allow for high-contrast visualization and realistic clinical settings, contrast agents are needed.

Flexible 3D-printed materials, particularly those fabricated using material jetting, are not always durable and can be sensitive to environmental factors [13, 17]. Some of the effects that can be observed in flexible 3D-printed models include liquid absorption, changes in flexibility over time, and structural weakness, particularly in thin parts. The uptake of contrast agents by the phantom material could cause mechanical instabilities and produce imaging artifacts. Application scenarios like stent graft testing, interventionalist training, and surgical planning might be negatively influenced by contrast uptake of the vessel phantom wall. For example, in MR or CT imaging, the phantom diameter would appear to be larger than it actually is, as the contrast agent could diffuse into the material wall, which interferes with quantitative measurements.

Thus, the aim of this study was to evaluate the contrast agent uptake behavior of two 3D-printed materials and a mix of these two when they are in contact with CT and MR contrast agents. The absorptive behavior of these materials is an indicator of their robustness and reusability in the context of using these 3D-printed materials in a perfused endovascular simulator.

## Methods

### 3D-printed cylindrical samples

A cylindrical sample with wall thickness of 2 mm, inner diameter of 20 mm, and height of 30 mm was designed in SolidWorks (Dassault Systèmes SolidWorks Corp., Waltham, MA, USA) to emulate a section of a young adult human aorta [18, 19]. The cylindrical samples were developed to be cup-like, so they can be filled with contrast agent solutions to test the absorptive properties of the materials used. The samples feature a flat-faced flange on the open end of the cup for easy placement inside a plexiglass sample rack. The .SLDPRT model was converted to an .STL file and imported into Stratasys printing software, GrabCAD Print (Stratasys Ltd., Eden Prairie, MN, USA) for material selection, support material selection, and print tray parameter selection.

**Fig. 1:**
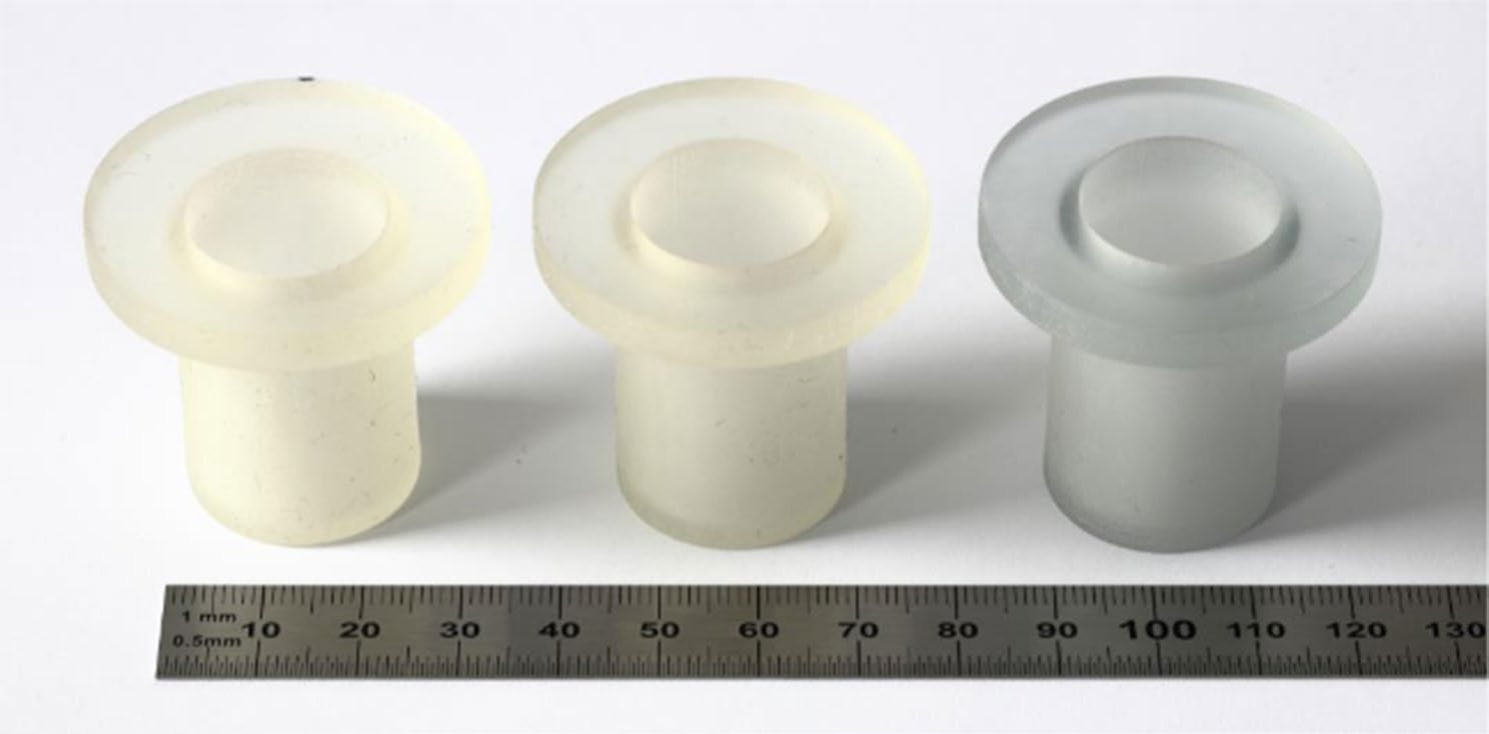
Cylindrical samples using different material blends. From left to right: Agilus, Agilus/VeroClear blend, VeroClear

The samples (Fig. 1) were printed using a Stratasys J850 Prime PolyJet 3D printer (Stratasys Ltd., Eden Prairie, MN, USA) with two different materials and one material blend. Using GrabCAD Print, the following materials were selected: Agilus30 (Shore hardness 30A) or VeroClear (Shore hardness 83d) (Stratasys Ltd., Eden Prairie, MN, USA). Additionally, these materials can be mixed through the material selection options in GrabCAD Print, thus allowing for numerous Shore hardnesses ranging between these two values. For this experiment, a Shore hardness of 70A was selected, referred to as “Agilus/VeroClear Blend” – a blend of VeroClear and Agilus (or its predecessor TangoPlus) is used frequently for 3D printing compliant vascular models [3]. Though a Shore hardness range of 30A to 50A has been shown to most accurately replicate aortic tissue, experiments to determine the most tissue-like material often do so using simple geometries, such as squares or dogbones [20, 21]. These results do not reflect the impact that support material removal has on the models – a high pressure water jet is used to remove the support material, which can cause rupture in vascular models [3]. Thus, 70A was selected as the median Shore hardness in this experimental group, as it is flexible yet robust.

The support material Stratasys FullCure 705 was used and was set to the lightest internal rigid grid “lite” in GrabCAD Print. This way, support material could easily be removed by hand, to avoid exposing the printed samples to water and caustic soda during support material removal, which are the standard methods of removal. The samples were printed two days before the start of the respective contact time, and support material was removed one day before the beginning of the contact time. This way, external influences, such as ambient humidity, were kept to a minimum and each sample received the same exposure time to air.

### Experimental setup

**Fig. 2:**
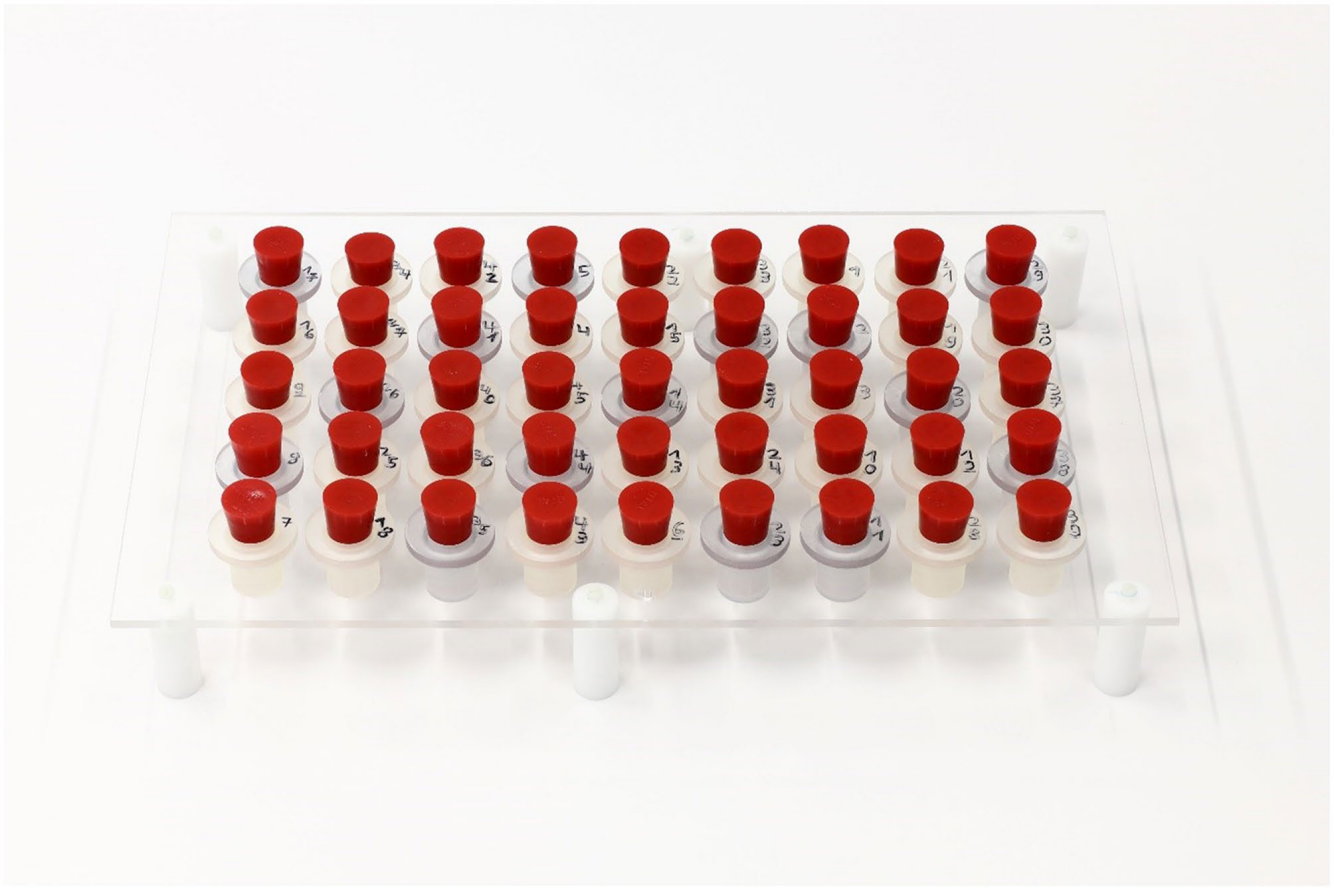
Experimental setup showing the plexiglass sample rack and cylindrical samples with red stoppers to seal the samples

Four variables were considered in the scope of this experiment: 3D-printed material, contact time (with the solution), contrast agent dilution, and imaging modality with corresponding contrast agent type (CT or MR). To determine the absorptive behavior of the samples, each sample was filled with 7.85 ml (fill height: 25 mm) contrast agent solution or saline solution, sealed with a rubber stopper, and placed in a sample rack. The sample rack allows precise placement of the samples for image evaluation and measurement (Fig. 2). Imeron 300 (Iomeprol) (Bracco, Milan, Italy) and Gadovist (Gadobutrol) (Bayer, Berlin, Germany) were the contrast agents used for CT and MR imaging, respectively. Three dilutions of each contrast agent were used: high, normal, and low, whereby “medium” represented a standard dilution that results in imaging characteristics comparable to a clinical setting [22, 23]. To dilute the contrast agents and act as a control solution, NaCl (0.9%) (B. Braun, Melsungen, Germany) was used. In Table 1 the dilution of each contrast agent is listed as a ratio.

**Table 1:** Dilutions for each contrast agent shown as a ratio of respective contrast agent to NaCl

Dilution Group	Iomeprol (CT)	Gadobutrol (MRI)
High	1:200	1:800
Medium (clinical)	1:50	1:200
Low	1:12.5	1:50

**Fig. 3:**
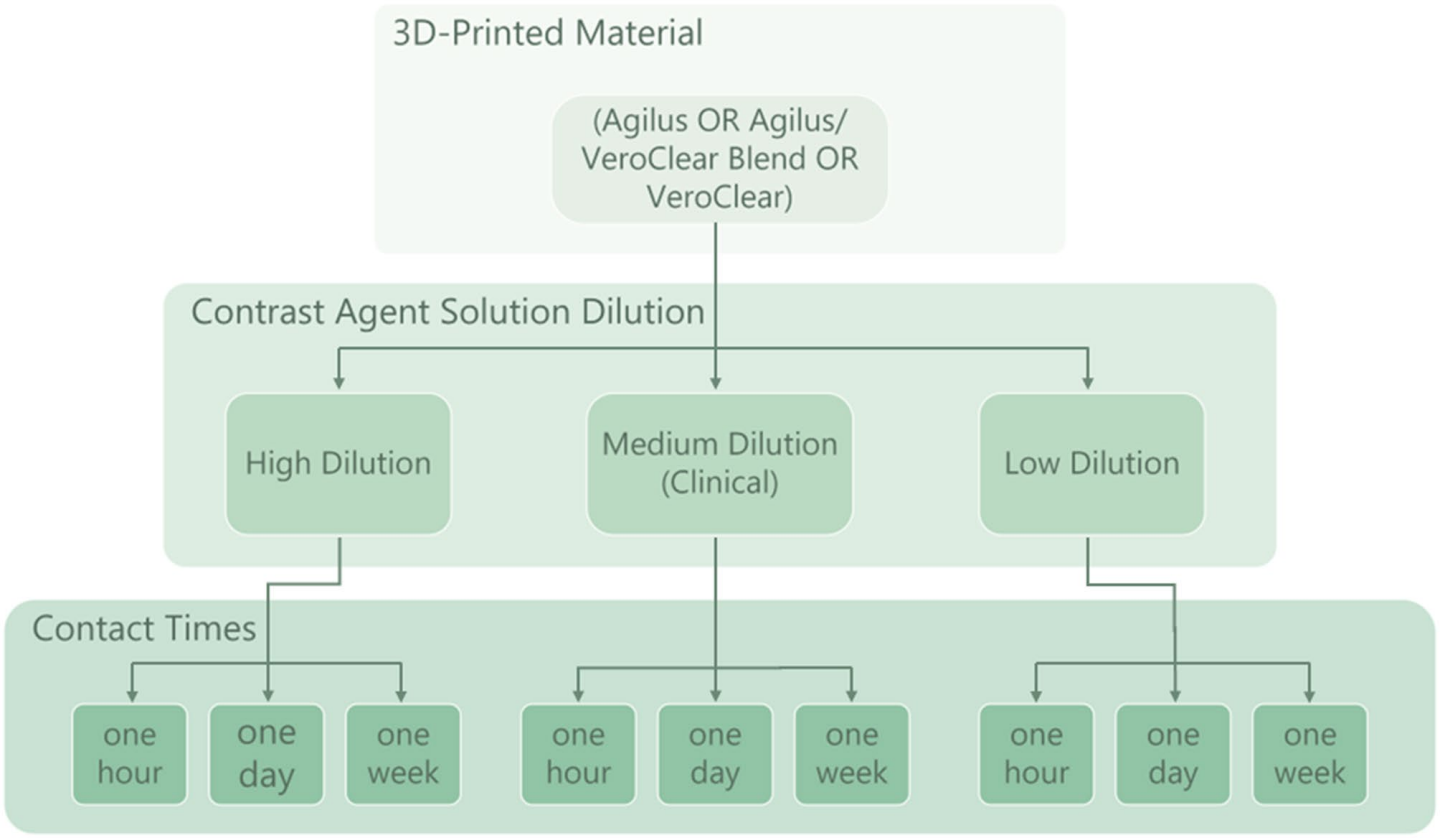
Summary of study parameters. Dilutions can be found in Table 1. Not shown here are reference samples

The samples remained filled with their respective solution for one of three contact times: one hour, one day, and one week. These contact times were selected as they represent realistic contrast agent-material interaction times for flow system experiments. The durations of the contact times were staggered so that all contact time groups concluded simultaneously. This way, all samples could be imaged at once. All samples remained in the sample rack during the entire duration of the exposure to the solutions, and were only removed once daily to gently shake the samples, to prevent sedimentation of the components of the contrast agents. The sample rack and samples were housed in a plastic tub with an airtight lid at a constant temperature of 21 °C. An overview of the variables tested for one contrast agent type is shown in Fig. 3.

### Relative change in mass

To determine if the samples absorbed the solutions to any degree, samples were weighed. The measurement method for determining the relative change in mass is based on the ASTM standard D57098 for analyzing water absorption in polymers and has been modified according to the specific requirements of this experiment. Before filling the samples with the solutions, all samples were weighed and the initial weight was recorded. After the CT and MR imaging was completed, all samples were emptied and carefully wiped out with a paper towel. The samples were then weighed once again. The relative change in mass was calculated and documented.

### CT and MR imaging

Before imaging, the sample rack containing all the samples was placed in a plastic container filled with eight liters of saline solution. This allows for better imaging quality of the materials and their surroundings and thus easier measurement. Samples were filled with contrast agent during imaging, as this represents a realistic scenario in which a 3D-printed vessel model is filled with contrast agent and is subsequently imaged.

CT imaging was performed in a clinical scanner (Brilliance iCT 256, Philips, Eindhoven, Germany) using following parameters with the aim to detect any potential contrast agent uptake of the sample wall: tube current time product = 625 mAs, tube voltage = 140 kV, slice thickness = 0.67 mm, total collimation width = 80 mm, pitch = 0.2, tube rotation time = 2.5 s, FOV = 253 mm.

Afterwards, the container with the samples was placed in the center of a clinical whole-body MRI-scanner (Magnetom Vida, 3T, Siemens, Erlangen, Germany). A 20-channel body coil was used and placed over the container. A T1-weighted turbo spin echo sequence was applied with following parameters: TE: 14 ms, TR: 855 ms, Flip Angle: 176°, FOV 300 × 300 mm, acquired matrix 512 × 384, slice thickness 2 mm, voxels interpolated to 0.6 × 0.6 ×2 mm.

### Image analysis and statistical evaluation

**Fig. 4:**
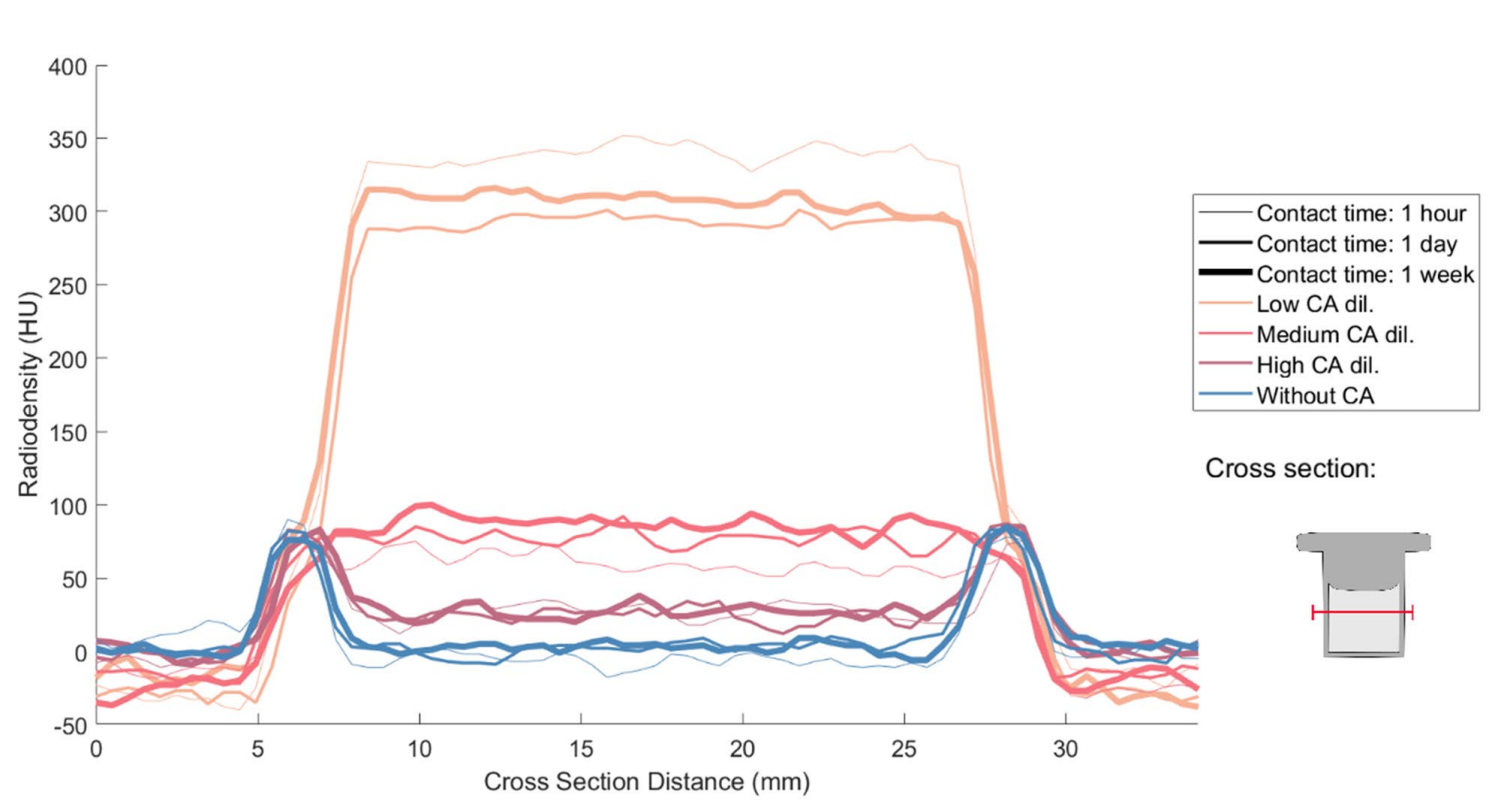
Intensity profiles for all contact times and contrast agent dilutions for Agilus samples. Below the legend, a schematic of the cross-section distance (red line) through the contrast-agent filled sample is shown

The density changes of the tomographic images, shown in an exemplary line plot for CT (Fig. 4), reveal a clear distinction between the lumen of the samples and the surrounding medium, in most cases. However, it is not possible to delimit the sample walls numerically from this data in a reliable manner.

**Fig. 5:**
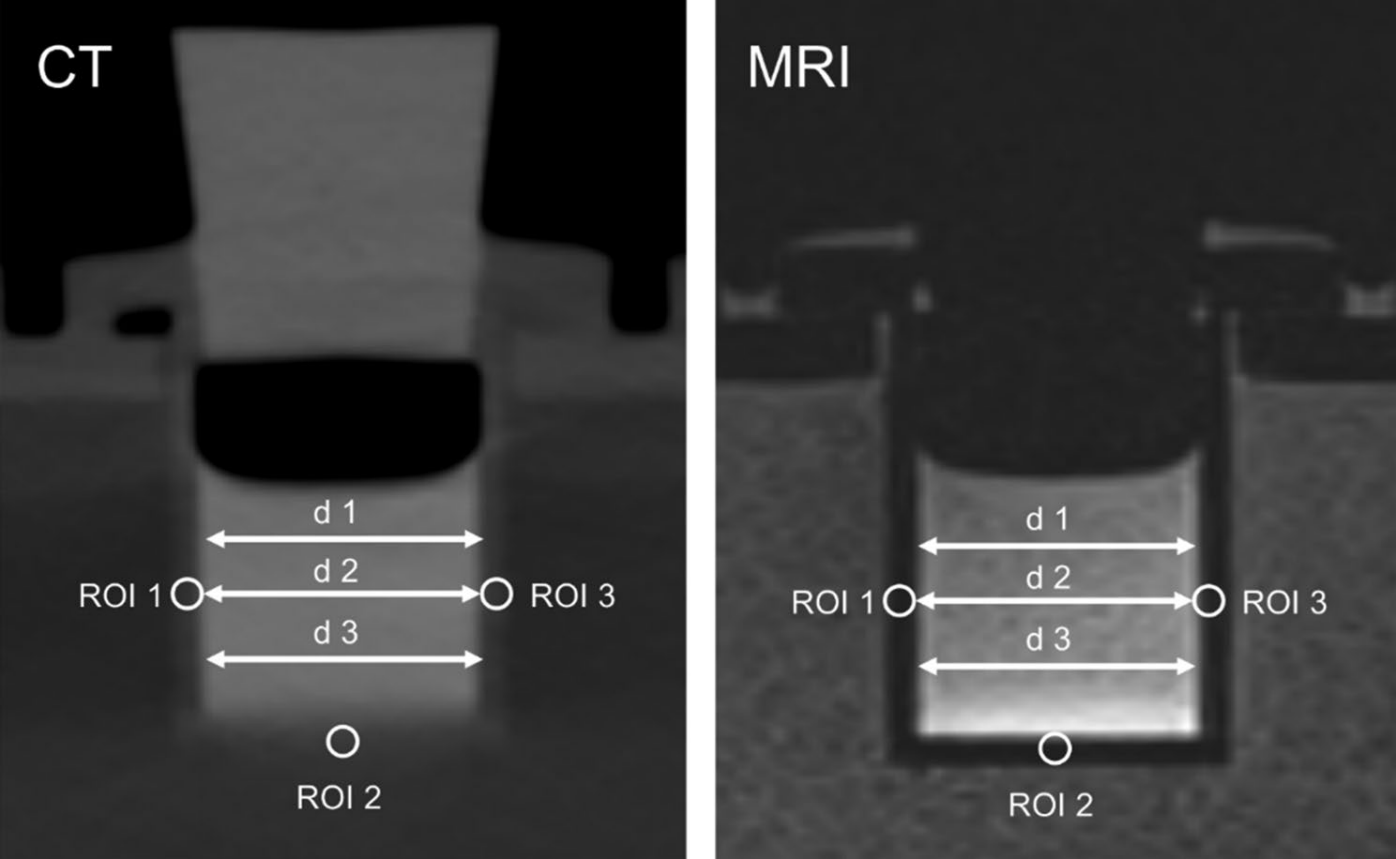
Measurement localizations for determining the change of diameter (d1, d2, d3) and density/signal intensity (ROI 1, ROI 2, and ROI 3) for detecting a potential contrast agent uptake of the sample walls

The image data were quantitatively evaluated by two board-certified radiologists (M.J.B. 5 years of experience, F.W. 8 years of experience). To accurately evaluate the contrast agent uptake throughout the entirety of the sample, the luminal diameters of the samples were determined at three defined points, as absorption into the wall would cause a diameter increase (Fig. 5). Furthermore, the contrast agent uptake might increase the density/signal intensity of the sample walls. Therefore, measurements at three defined locations within the sample wall have been performed in the coronary tomographic images (Fig. 5). According to the nominal wall thickness of the phantoms, the region of interest (ROI) was defined as a circle with a 2 mm diameter. For all measurements, the slice with the largest area of the depicted sample was selected. Both investigators performed their measurements blinded from one another on identical images. The measurements were carried out at a clinical radiology workstation using DeepUnity Diagnostic software (DeepUnity Diagnost, Version 1.2.0.3, Dedalus S.p.A.,). All measurements from both observers for each location (diameter, density (in Hounsfield units)/signal intensity (in arbitrary units)) are displayed as mean and standard deviation (SD).

### Statistical evaluation of contrast agent uptake

The data was evaluated for normality using the Shapiro-Wilk Test to determine which statistical test is most apt for further evaluation. Based on these results, a generalized linear mixed model (GLMM) was used to investigate the effects of material, contrast agent dilution, contact time, and their impact on the diameter or radiodensity/signal intensity of the 3D-printed cylindrical samples. More specifically, the three variables are grouped in groups that are independent of the experimental design to discern the effect *each variable* has independently on the 1) signal intensity (a.u.) or radiodensity (HU) 2) diameter of the sample. The goal of this evaluation is the determination of an ideal material (among those tested) that can be recommended for use in 3D printed vascular models that are perfused with solutions laden with contrast agents. A GLMM was specifically selected as an evaluation method due to the low number of data points and heterogeneity of the data.

The calculation was performed using the lmer function from the lmerTest package in R (R Version 4.4.0, http://www.r-project.org) considering the fixed effects of the factors and a random effect for the subjects. The restricted maximum likelihood criterion was calculated for the evaluation, and the random effects were reported as variances and standard deviations. The *t*- and p-values were extracted from this evaluation and were reported. The graphical processing of the data was performed by using MATLAB (The MathWorks Inc, Natick, MA, USA).

### Interrater reliability

An intraclass correlation coefficient (ICC) with 95% CI was used to determine the reliability of measurements between the two observers. For this study, the “consistency” model was preferred over the “absolute agreement” model because all participants were evaluated by the same radiologists and because systematic differences between these radiologists were irrelevant. The ICC values were calculated based on a two-way random effects model with the statistics software Jamovi (The jamovi project, 2024, Version 2.5, Sydney, Australia). The ICC values (type absolute agreement) can be interpretated as follows: < 0.5 poor agreement, 0.5 > 0.75 moderate agreement, 0.75 > 0.9 good agreement, > 0.9 excellent agreement, respectively [20].

## Results

### Mass changes of the samples over time

**Fig. 6:**
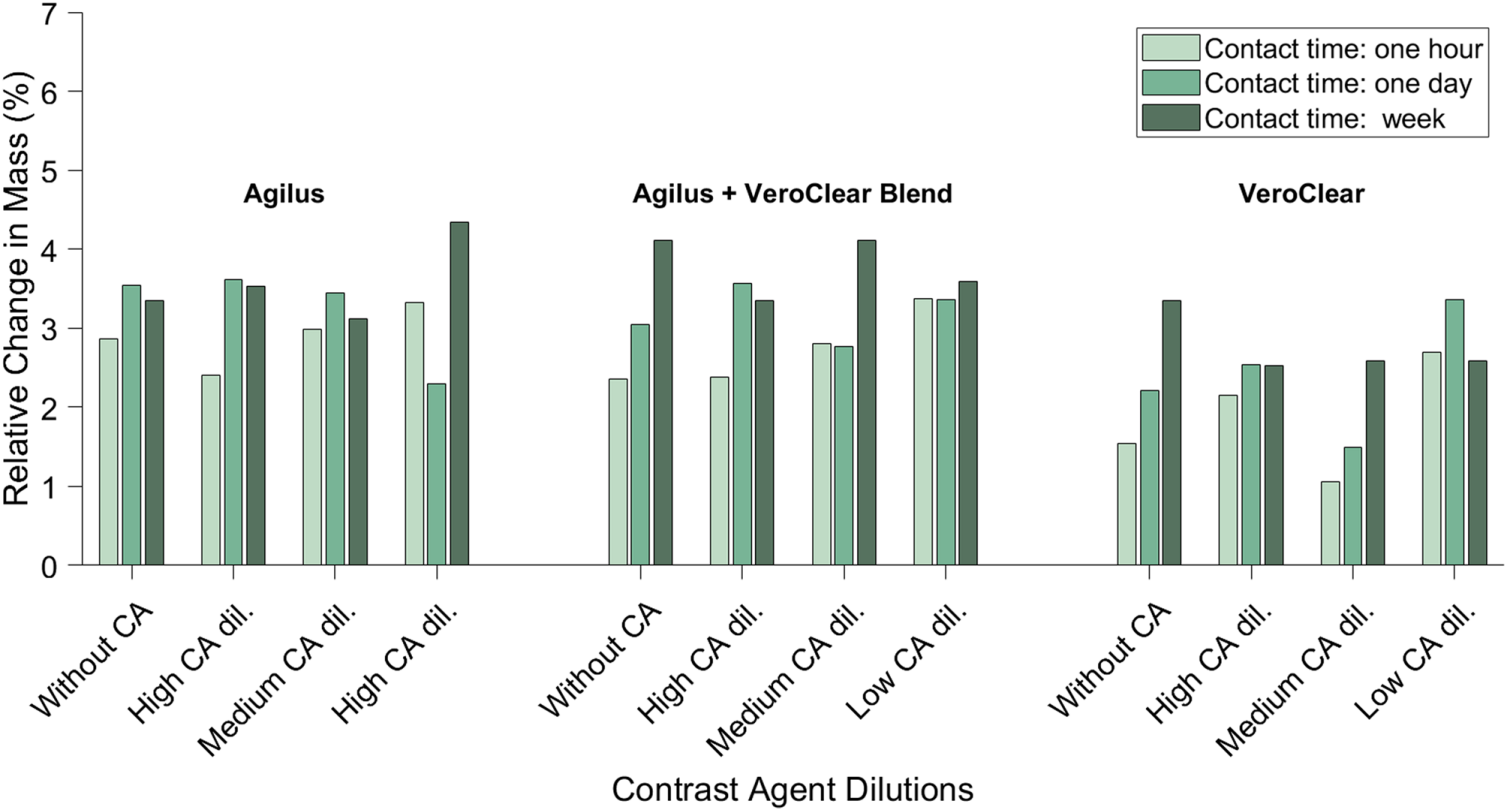
Overview of the relative mass increase after one hour, one day, and one week. The results are grouped for each 3D printing material we tested. The dilutions can be found in Table 1

All samples exhibited an increase in mass after at least one hour of contact with the solution (either without contrast agent or with diluted contrast agent). The smallest increase after one hour (1.1%) was observed in VeroClear with a medium dilution of contrast agent, while the largest (2.9%) was in Agilus without contrast agent. After one week of contact, the smallest mass increase was 2.5% (VeroClear, both middle and low contrast agent dilutions), and the highest was 4.4% (Agilus, lowest contrast agent dilution). Overall, a general trend of increasing mass over time was evident in most samples, as can be seen below for all 3D-printed materials in Fig. 6. Among the three materials tested, Agilus demonstrated the largest mass increase, whereas VeroClear exhibited the smallest increase throughout our experiments.

### Interrater reliability

The interrater reliability of diameter and signal intensity measurements performed on MR images was moderate. Measurements of diameter in CT images had moderate interrater reliability, and density measurements had a good interrater reliability. ICC values can be found in Table 2.

**Table 2:** Intraclass coefficients for diameter and signal intensity or density values in MR or CT images of 3D-printed samples

Imaging Modality	Values Measured	ICC (95% CI)	Interpretation
MRI	Diameter	0.60 (0.48 to 0.70)	moderate
	Signal intensity (a.u.)	0.59 (0.47 to 0.69)	moderate
CT	Diameter	0.60 (0.48 to 0.70)	moderate
	Density (HU)	0.79 (0.72 to 0.85)	good

### Statistical evaluation of contrast agent uptake

Normality tests showed that the diameter and radiodensity measurements of the CT are not normally distributed. However, the diameter measurements of the MR images showed a normal distribution, while signal intensity measurements did not show a normal distribution.

A GLMM was used to determine the effect of each of the variables (material, contact time, or contrast agent dilution) on the diameter of the samples or radiodensity/signal intensity in the sample walls, respectively. Heightened t-values and estimates indicate increased diameter or signal intensity/radiodensity. Low t-values and estimates indicate lesser diameters or signal intensity/radiodensity when compared to the control group. An analysis was performed without exclusion of outliers. The reference values (control values) stem from the following sample: Agilus, no contrast agent, contact time: one hour before measuring. The estimated mean diameter of the reference sample is 19.99 mm.

#### CT: Interaction between 3D-printed materials and sample diameter

**Fig. 7:**
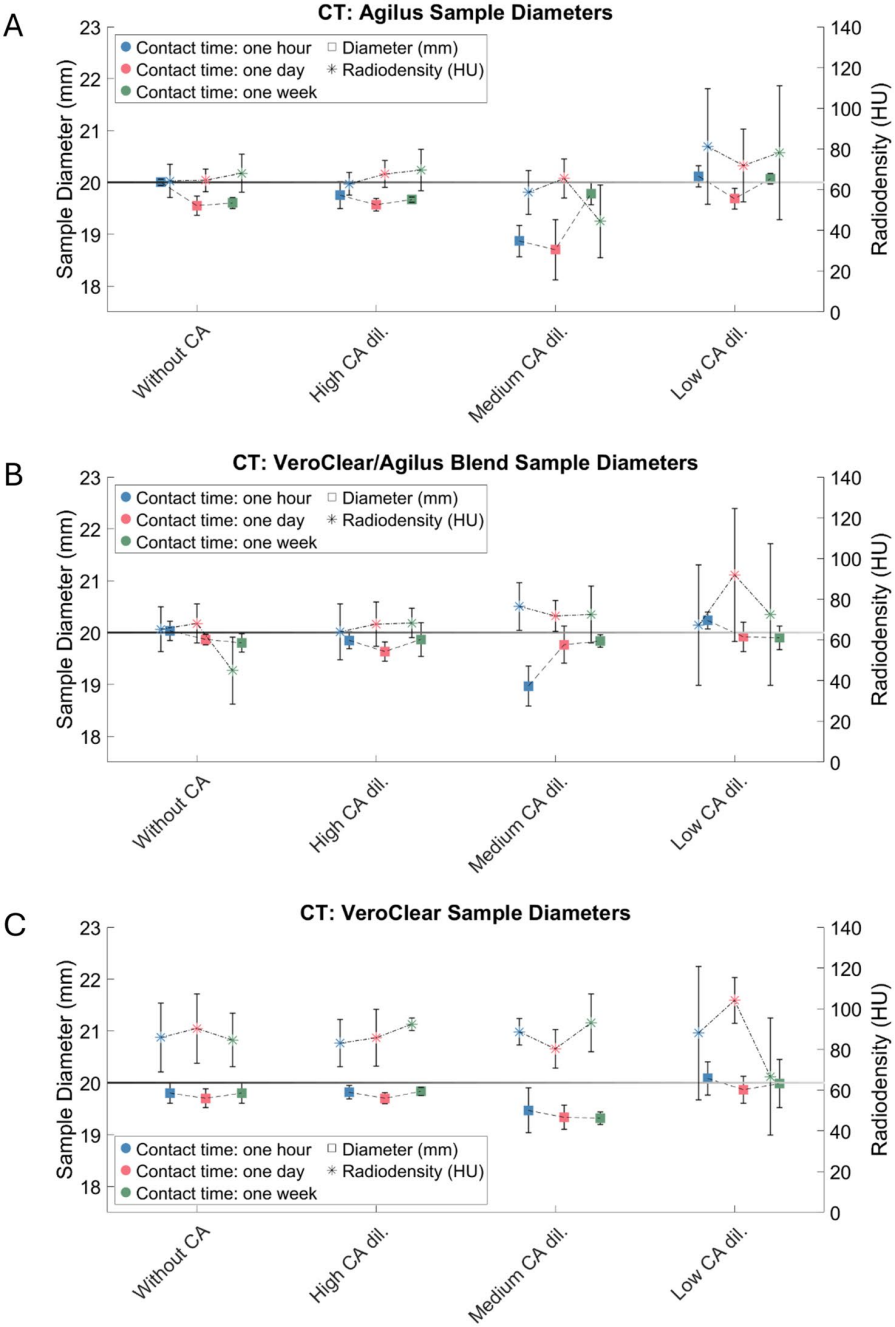
Changes in measured diameter and density of the sample walls in CT images. The samples were printed from Agilus (**A**), Agilus/VeroClear Blend (**B**) or solely VeroClear (**C**). The black horizontal line indicates the nominal sample diameter of 20 mm

Material blend (VeroClear/Agilus) (*t* = −0.51, *p* = 0.61): No significant effect compared to pure Agilus (reference value) (Fig. 7). The material blend (VeroClear/Agilus) did not substantially affect the depicted diameter. VeroClear (*t* = −2.31, *p* = 0.02): Significant negative effect when compared to the reference value. This means that the images of VeroClear samples had a larger diameter than the reference material sample.

#### CT: Interaction between 3D-printed materials and radiodensity

The material blend VeroClear/Agilus revealed no significant effect with respect to an increase in radiodensity in the sample walls (*t* = 0.75, *p* = 0.45) (Fig. 7). VeroClear samples (*t* = 3.40, *p* < 0.001) showed a significant result and thus a higher radiodensity than the reference material Agilus.

#### MRI: Interaction between 3D-printed materials and sample diameter

**Fig. 8:**
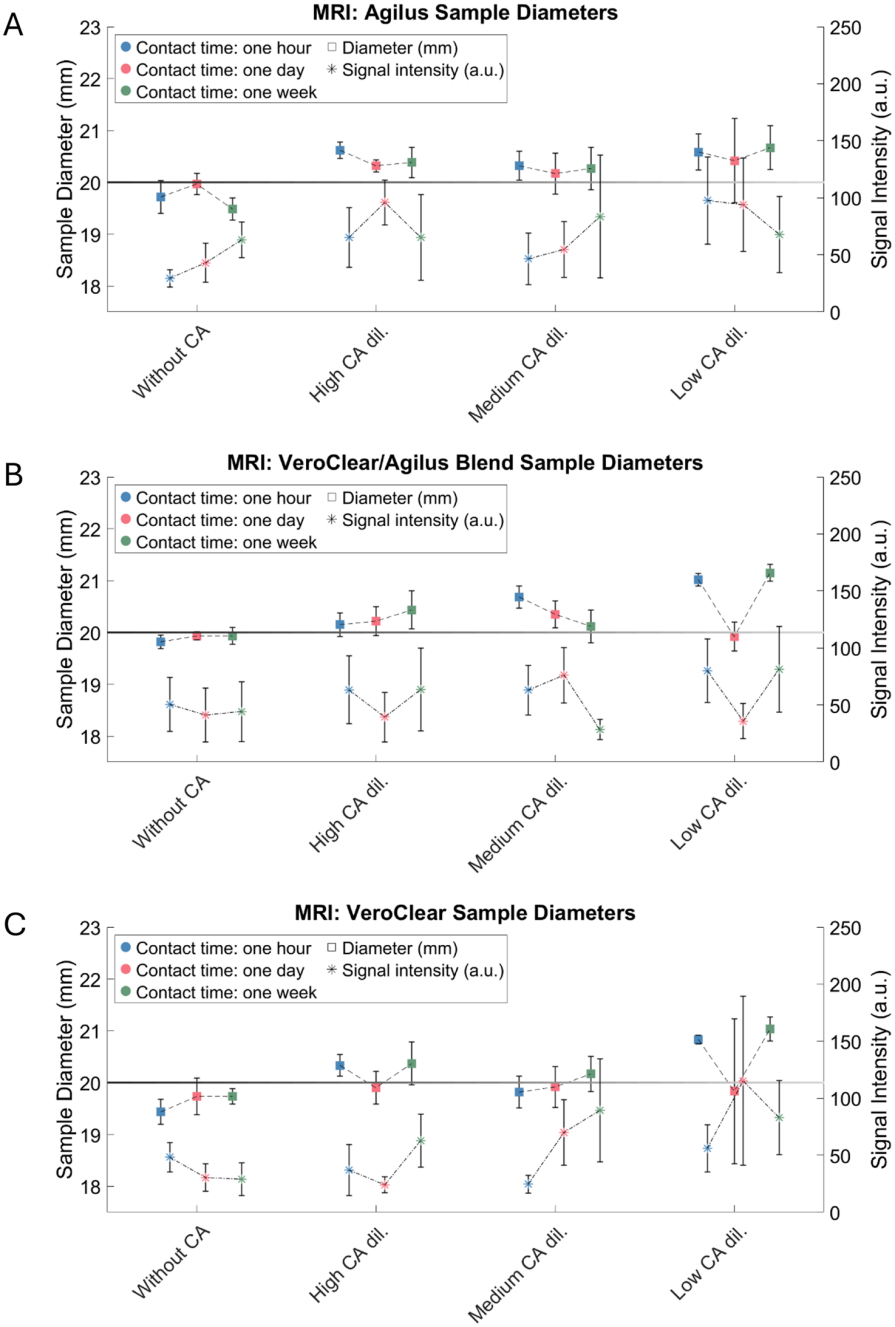
Variations in measured diameter and signal intensity of the sample walls in MRI. The samples were printed from Agilus (**A**), Agilus/VeroClear Blend (**B**) or solely VeroClear (**C**). The black horizontal line indicates the nominal sample diameter of 20 mm

The material blend VeroClear/Agilus samples (*t* = 0.83, *p* = 0.41) and VeroClear samples (*t* = −1.49, *p* = 0.14) showed no significant effects (Fig. 8).

#### MRI: Interaction between 3D-printed materials and signal intensity

In this analysis, the material blend samples VeroClear/Agilus (*t* = 0.21, *p* = 0.83) and VeroClear samples (*t* = 0.21, *p* = 0.81) revealed no significant effects (Fig. 8).

## Discussion

3D-printing materials used for vascular phantoms did not take up a detectable amount of CT and MRI contrast agents under clinical imaging conditions in the evaluated timeframes. A general mass gain over time, albeit small, must be reported.

In addition to human-like morphology and mechanical characteristics [24], the temporal stability of vascular phantoms during extensive experiments is of highest interest. Especially changes in the depicted diameter of the phantoms could negatively influence interventional instrument testing, like the evaluation of stent grafts. Our results indicate that the widely used 3D-printing materials, Agilus, VeroClear and the combination of both, are not strongly influenced by contrast agents in experimental imaging setups. Nevertheless, there was a relative mass increase of up to 4.4% observed in our study with the lowest contrast agent dilution. The mass gain is in line with the manufacturer information available for VeroClear. In the product data sheet, a water absorption rate of 1.1–1.5% is given for 24 hours [25]. In our study, we observed a relative mass gain of 1.5% (without contrast agent) and 2.7% (lowest contrast agent dilution) after 24 hours of contact time. For other widely used 3D-printing materials, the mass gain after water contact is stated to be in the same range [26].

There are different types of destruction for polymeric materials in the presence of solvents described: dissolution, delamination, disintegration and swelling [17]. In our work, we macroscopically did not observe any of them. Furthermore, the samples did not show any deformation during the experimental course, which is confirmed by our diameter measurements. In relation to the tested contrast agent dilutions, the total mass increase of up to 4.4% might be too small to cause detectable imaging changes of the printed samples.

The results of the diameter and density/signal intensity measurements and ICC ratings must be put into relation to the spatial resolution of the applied imaging modalities. The generated CT images had a submillimeter resolution (~0.6 mm). The MR images had a resolution in the range of 1 mm. To emulate a human aorta, a sample wall thickness of 2 mm and an inner diameter of 2 cm was selected [18, 19]. The relation between the small sample wall thickness and the resolution of the clinical CT- and MRI-scanner set the values of the interrater reliability into perspective. Furthermore, the statistical evaluation was performed to detect potential trends in the contrast agent uptake behavior of the tested materials so to determine an optimal material for this application. By taking the resolutions of the clinical scanners used in this experiment and the geometrical inaccuracies caused by manufacturing of the samples into account, the statistical results do not allow the priorization of a single material, nor confirm contrast agent uptake by the tested 3D-printed resins. Further testing with shorter contact time intervals could offer clearer insights on the interaction of the 3D-printed materials with contact time and contrast agent dilution.

This study has several limitations, which need to be discussed. Only two different materials and a combination thereof were investigated. Consequently, the portfolio of tested materials should be extended to more 3D printing polymers in the future. With regard to sample manufacturing, soft materials (Agilus) were used, which may cause deformation upon handling, which could lead to inaccuracies in diameter measurements. Additionally, the support material, which is visually very similar to the materials used, was manually removed from the samples. It is possible that residuals of the support material remained in the samples, skewing results. Furthermore, the experimental timeframe was limited to one week. Hence, effects which might occur during longer experiments are undetectable in this study. Numerous variables were evaluated in this study—to more precisely determine the interaction of a single variable with the printed materials used, separate investigations may be needed. A variation of the scanning parameters, such as T1- and T2-mapping in MRI, might detect changes in signal properties of the samples that have been neglected thus far. Regarding CT images, the presence of signal “blooming” cannot be completely eliminated and could be present to a small degree, though efforts were made to minimize it through using a higher tube voltage. Additionally, a tube voltage of 140 kV allows for higher quality images and less noise than lower tube voltages.

In summary, we cannot exclude that some of the 3D-printed materials tested in this work do absorb contrast agents to a small degree, though the contrast agent uptake is not detectable with clinical imaging methods. Consequently, the tested 3D-printed materials are robust enough for reuse and experimentation with contrast agent solutions for clinical imaging experiments.

## Data Availability

The raw data supporting the conclusions of this article will be made available by the corresponding author, without undue reservation, to any qualified researcher.

## Abbreviations

CT: Computer Tomography
CA: Contrast Agent
GLMM: Generalized Linear Mixed Model
ICC: Intraclass Correlation Coefficient
MRI: Magnetic Resonance Imaging
